# Online case-based learning to enhance pediatric endocrinology resident education

**DOI:** 10.1186/s12909-025-08517-5

**Published:** 2026-01-08

**Authors:** William Freeman, Andrew Kanouse, Graeme R Frank

**Affiliations:** 1 Department of Pediatrics, Cohen Children’s Medical Center, Donald and Barbara Zucker School of Medicine, Hofstra University, 269-01 76th Avenue, New Hyde Park, New York, USA; 2 Division of Endocrinology, Department of Pediatrics, Cohen Children’s Medical Center, Donald and Barbara Zucker School of Medicine, Hofstra University, 1991 Marcus Avenue St. M100, New Hyde Park, New York, 11042 USA

**Keywords:** Medical endocrinology, Pediatric endocrinology, Online module, Resident education, Medical education, Case-based learning

## Abstract

**Background:**

While much of medical resident trainee exposure to pediatric endocrinology is geared towards hospital-based management of hyperglycemic urgencies and emergencies, the American Board of Pediatrics certification exam and trends within pediatric endocrinology referrals require an understanding of more nuanced aspects of the endocrine system that include abnormalities of growth, puberty, sexual differentiation, and the thyroid gland, which are less often encountered in emergency, inpatient, and critical care settings. Challenges in medical education include increased direct faculty supervision requirements and heightened demand for clinical productivity among faculty at the expense of time for teaching. In response, online case-based learning (CBL), which utilizes real or simulated clinical scenarios with high rates of flexibility and learner satisfaction, offers a viable learning option. This study sought to determine whether an online CBL tool would contribute to resident learning during an outpatient pediatric endocrinology elective rotation.

**Methods:**

Clinical cases reflecting different scenarios within pediatric endocrinology, including growth, puberty, thyroid disorders, sexual differentiation, and calcium metabolism, were created in Microsoft PowerPoint with an audiovisual recording created using TechSmith Camtasia Studio and were made available online. Each case posed several questions, each followed by a discussion and rationale for the correct answer. Trainees were encouraged to complete the CBL modules prior to or early during their pediatric endocrinology rotation. After completion of the elective rotation, residents were sent a questionnaire via SurveyMonkey to evaluate the CBL modules based on the Kirkpatrick model.

**Results:**

A total of 29 residents participated in the evaluation. Resident rating of satisfaction of cases was 76% ‘a lot’ and 24% ‘some’. Resident rating of knowledge acquisition was 79% ‘a lot’ and 21% ‘some’. Resident rating of application of learning was 79% ‘a lot’ and 21% ‘some’. Finally, resident rating of impact on clinical practice was 62% ‘a lot’ and 38% ‘some’. No categories included responses in the “a little” or “none” options and free-text comments were overwhelmingly positive.

**Conclusion:**

This evaluation supports that an online CBL model of pediatric endocrinology education can be an effective adjunct to the current state of medical education. Overall, utilization of CBL education bolstered resident education in critical areas of endocrinology during their outpatient rotation as measured by self-reported metrics. Similar online CBL models can promote competence, confidence, and interest in pediatric endocrinology and other domains of medical education both for expansion of interest in the field of pediatric endocrinology itself and confidence of general pediatricians in approaching it.

**Supplementary Information:**

The online version contains supplementary material available at 10.1186/s12909-025-08517-5.

## Background

In United States-based pediatric medical residency programs, pediatric endocrinology is commonly offered as an optional elective rotation not required for completion of training. As such, much of resident exposure to pediatric endocrinology focuses on management of inpatient hospital-based emergencies/urgencies, most commonly those of a hyperglycemic nature (i.e. diabetic ketoacidosis and/or hyperglycemic hyperosmolar non-ketotic syndrome), with fewer residents experiencing outpatient pediatric endocrinology. This comes with the potential to neglect a significant contingent of pediatric endocrinology exposure, including abnormalities of growth, puberty, and the thyroid gland that ultimately account for most new outpatient endocrinology referrals across much of the Western world [1, 2]. Additionally, while approximately 3% of the American Board of Pediatrics certification examination is explicitly geared towards the domain of pediatric endocrinology, a number of other topic domains refer to material learned through exposure to pediatric endocrinology not accounted for in this percentage, such as normal puberty, gender-affirming care, ambiguous genitalia, menstrual abnormalities, and obesity-topics found within the adolescent, neonatal, genitourinary, and well-child care domains [3]. This is concerning for a lack of resident experience in outpatient pediatric endocrinology coupled with professional expectation to understand outpatient pediatric endocrinology. Bridging this gap requires dedicated medical education.

Medical education continues to face challenges including decreased protected time for senior clinical teaching, secondary to increased productivity demands on faculty, as well as increased requirements for direct attending supervision, at times at the expense of resident autonomy and progressive independence [4–6]. The shift toward increased direct supervision, while intended to enhance patient safety and quality metrics of medical care, may hinder the development of independent clinical judgment, trainee drive toward self-driven learning, and overall knowledge acquisition [5, 6]. These factors have diminished experiential learning and opportunities for easy-to-perform, frequent formative feedback and guidance. This is particularly important in areas of medical education that receive less rigorous dedicated exposure-such as pediatric subspecialties-where there may be a perceived ability to forfeit focus on the intricacies of these specialties for the sake of learning a minimum amount necessary to pass standardized certification examinations.

In response, educational innovations such as online case-based learning (CBL) have gained traction. CBL leverages real or simulated clinical scenarios to foster active learning, clinical reasoning, and knowledge integration [7–10]. The COVID-19 pandemic accelerated the adoption of technology-enhanced and remote learning modalities, with evidence ultimately supporting the effectiveness, flexibility, and learner satisfaction associated with online CBL [7, 11–14]. A meta-analysis from Zhang et al. [8] indicated that CBL is a more effective strategy for improving knowledge and clinical skills among medical students compared to lecture-based learning and flipped classrooms. Studies, however, remain limited regarding the utilization of such modalities in pediatric subspecialties for trainees that are expected to have acquired this knowledge by the end of their residency for successful completion of certification exams and whether CBL would be a useful adjunct for such subspecialties that trainees may be less familiar with.

This study aimed to evaluate whether directed, online CBL modules in pediatric endocrinology, a subspecialty that remains an optional but not mandatory aspect of pediatric residency training, could effectively teach a broad array of high-yield concepts to residents. The study assessed self-appraised knowledge gain and learner perceptions of the modules’ design to enhance education during a pediatric endocrinology rotation. The hypothesis was that online CBL, independent of in-person encounters, would improve both knowledge retention and rotation experience and thereby strengthen pediatric competency.

## Methods

All residents rotating through the Division of Pediatric Endocrinology and Diabetes at Cohen Children’s Medical Center were eligible to participate. Typically, 1–2 of the program’s approximately 90 total pediatric residents rotate through the elective each month. Included in this study were the residents rotating over a 2.5-year time span and consisted of first, second, and third year pediatric residents. Before the rotation, residents received an email inviting them to complete five self-directed online CBL modules prior to their first day with the aim of bolstering this knowledge as they progressed through the elective. The information presented was uniform across year of training. Given respondent anonymity within the context of an educational study, this research was exempt from IRB review.

**Fig. 1:**
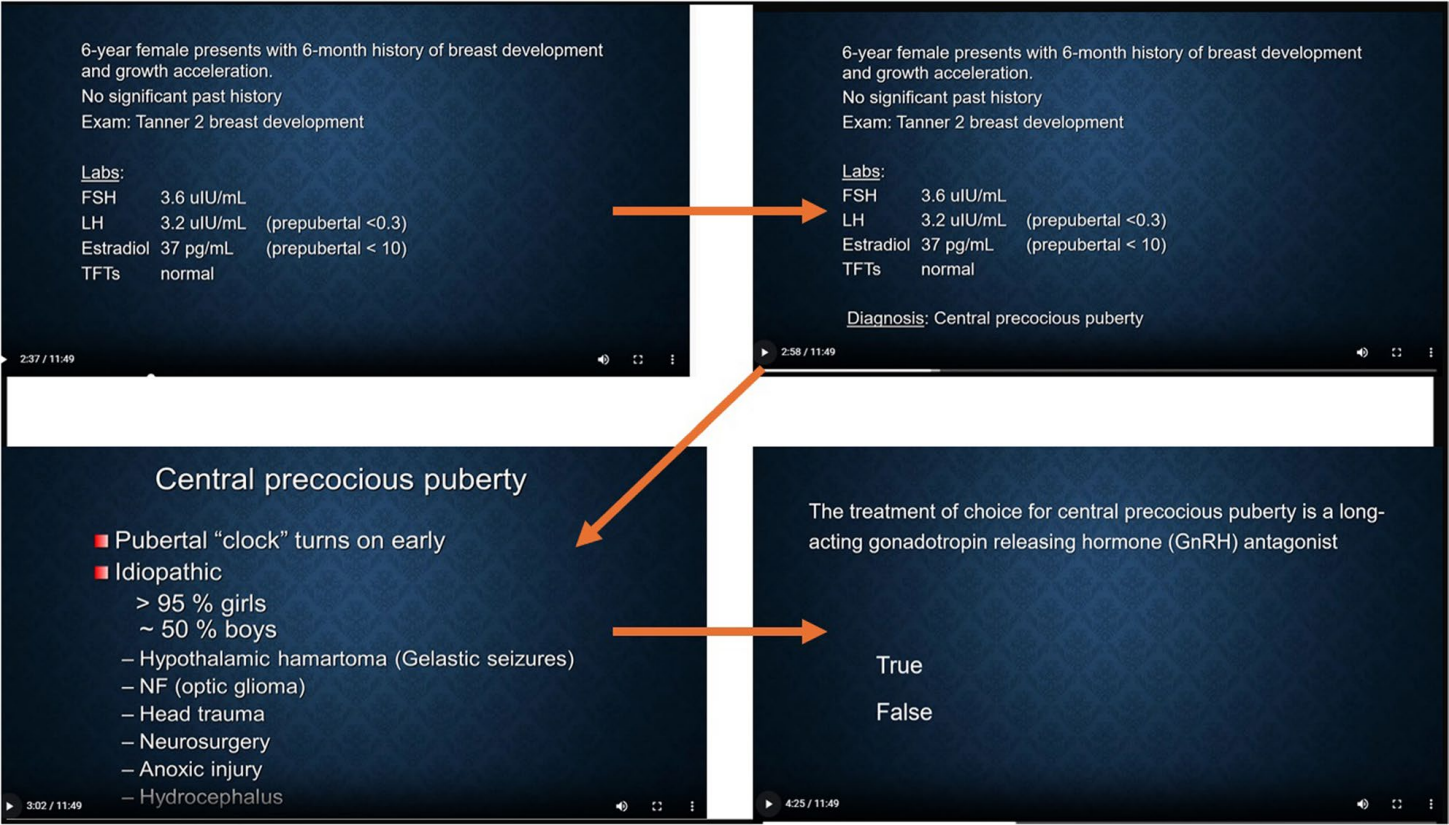
A case is posed in the puberty module with an audiovisual discussion and a follow-up knowledge-acquisition evaluation (arrows for visual guidance on flow). ^*^FSH (Follicular Stimulating Hormone) 3.6 uIU/mL (normal reference range (NRR) for age 0.72–5.33 uIU/mL); LH (Luteinizing Hormone) 3.2 uIU/mL (NRR < 0.3 uIU/mL); Estradiol 37 pg/mL (136 pmol/L; NRR < 10 pg/mL)

**Fig. 2:**
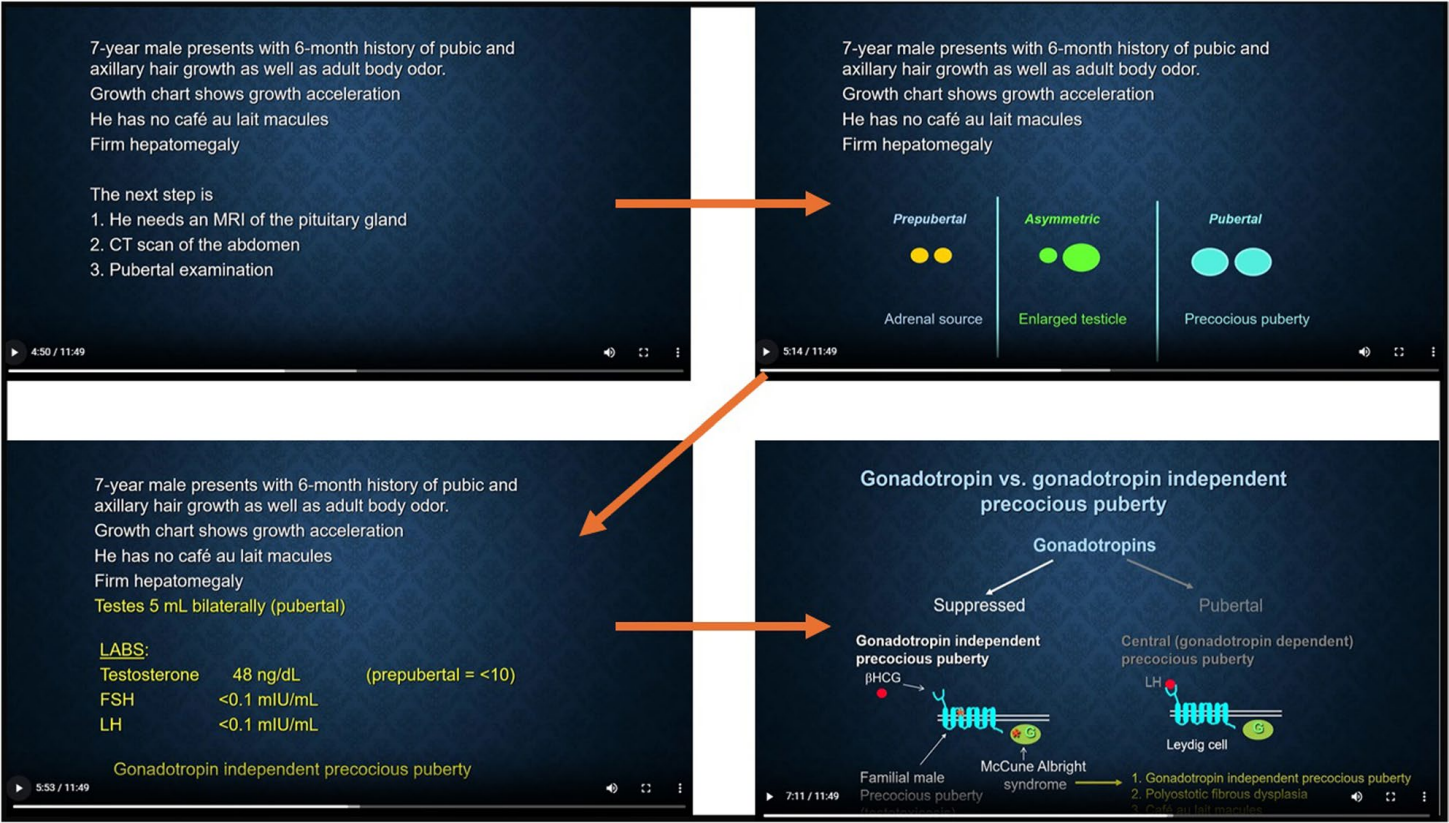
A second example of a case-based question posed in the puberty module followed by an audiovisual explanation (arrows for visual guidance on flow). ^*^Testosterone 48 ng/dL (166 nmol/L; NRR for age < 10 ng/dL); FSH < 0.1 uIU/mL (NRR 0.21—4.33 uIU/mL); LH (Luteinizing Hormone) < 0.1 uIU/mL (NRR < 0.3 uIU/mL)

**Fig. 3:**
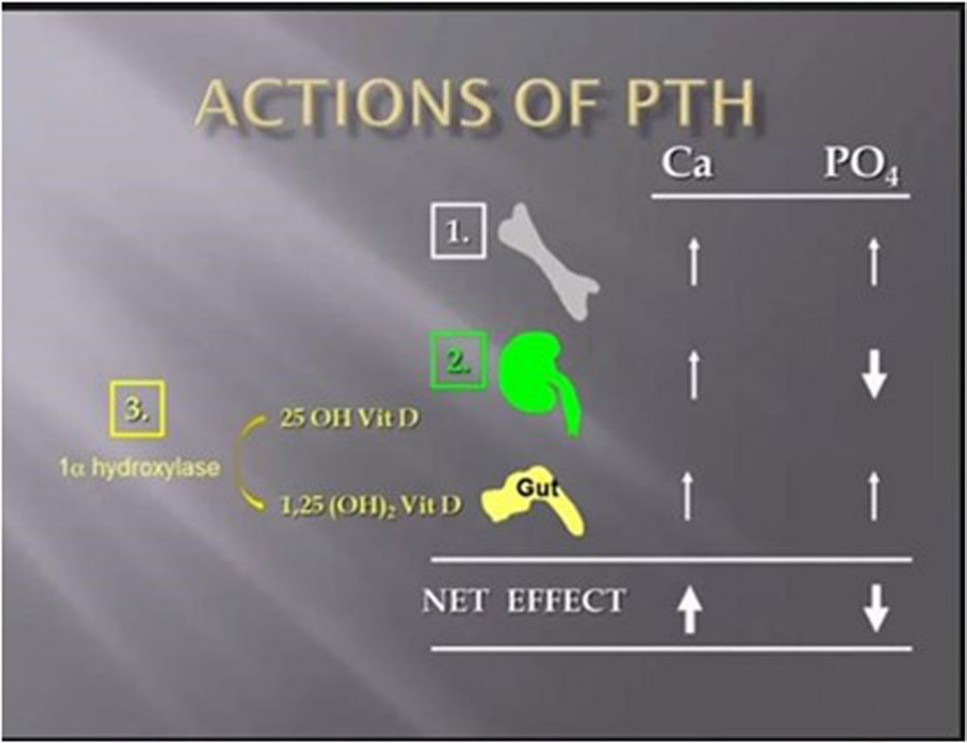
Example of the rationale of a knowledge-based acquisition question provided via audiovisual recording from the calcium module

Modules, developed in Microsoft PowerPoint, addressed five core pediatric endocrinology topics: growth, puberty, thyroid disorders, sexual differentiation, and calcium metabolism. Modules ranged from 5 to 20 min. Using TechSmith Camtasia, cases were recorded and converted into interactive audiovisual files for online access. Clinical histories were followed by sequential probing questions requiring responses to advance. Figs. 1 and 2 as representation demonstrate the clinician’s approach to precocious puberty, a commonly encountered endocrinopathy critical for pediatric clinician education. Immediate feedback with animated audiovisual explanations of mandatory questions was provided for each as illustrated in Fig. 1. Fig. 3 similarly demonstrates feedback that was included following a question relevant to the calcium metabolism education provided immediately prior. Questions included multiple-choice and short-response formats.

After the elective was finished, residents were asked to complete a four-item voluntary, anonymous SurveyMonkey questionnaire evaluating the modules via a Likert scale aligned with the four levels of the Kirkpatrick model: reaction (satisfaction), learning (knowledge acquisition), behavior (application in practice), and results (perceived impact) [15–19]. Additionally, residents were given free text comment space associated with each of the four questions. This questionnaire can be found in Supplementary File 1. This data was analyzed and graphed with SurveyMonkey and Excel.

## Results

**Fig. 4:**
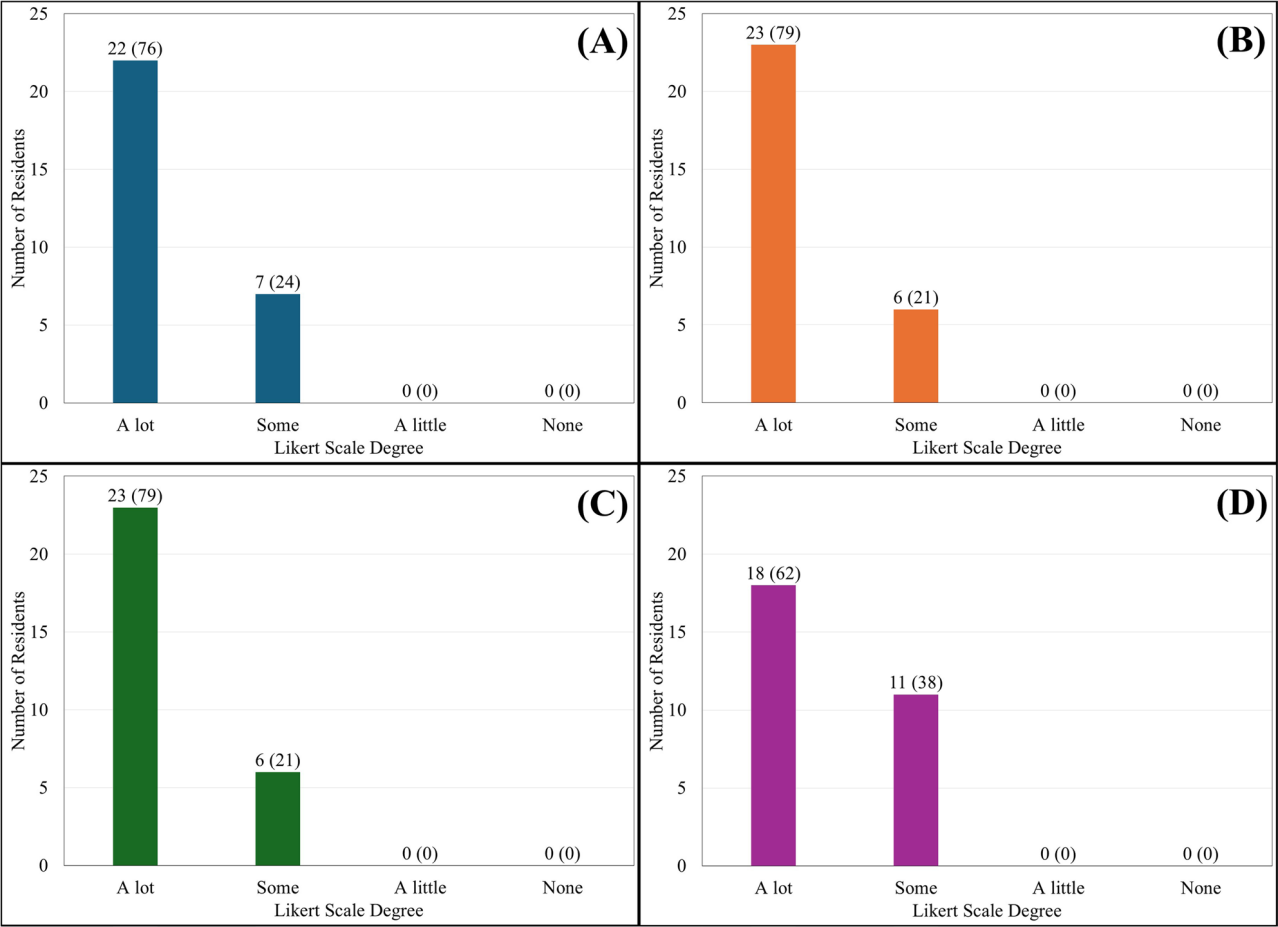
Resident Likert responses. ^*^n = 29, with values expressed as total (percentage). **A** Did I enjoy the audiovisual modules? **B** Did I increase my knowledge and learning as a result of doing these interactive audiovisual modules? **C** Will I be able to use the information I learned from these modules when evaluating children with various conditions such as short stature, early or delayed puberty, abnormal thyroid function, etc.? **D** Do I think that the knowledge and learning will improve my effectiveness as a pediatrician?

There were 29 of 32 eligible residents who participated during the 2.5-year period analyzed. Resident rating of satisfaction with cases was 76% (*n* = 22) ‘a lot’ and 24% (*n* = 7) ‘some’. Resident rating of learning was 79% ‘a lot’ (*n* = 23) and 21% ‘some’ (*n* = 6). Resident rating of application in practice was 79% (*n* = 23) ‘a lot’ with 21% (*n* = 6) ‘some’. Lastly, resident rating of perceived impact was 62% (*n* = 18) ‘a lot’ and 38% (*n* = 11) ‘some’. There were no low ratings (‘a little’ or ‘none’) on any of the four areas (Fig. 4). Free text comments in all four areas were uniformly positive (Table 1).

**Table 1:** Resident free text comments

1. Enjoyment: Did I enjoy the enjoy the audiovisual modules?
I appreciate that the videos covered high yield points in a clear and succinct manner
I thought they were very helpful- would even want more!
Appreciate having relevant material to review at the beginning of the elective
Cases were interesting and relevant to what I was seeing in clinic
Facts conveyed in a concise and interactive way
The modules were an appropriate duration and intensity that allows for learning without making the assignment stressful or very time consuming
Yes, the cases were nicely presented, and had great transition with interactive questions that made it engaging and highlight key concepts to take away. Very informative!
I enjoyed working through the cases and found them to be concise while covering enough breadth and depth. The interface itself was not that user friendly
The case scenarios make it more memorable and relatable. The animations were also a nice touch.
I appreciate the helpful slides explaining the more complicated ideas.
I rewatched each video a few times as they significantly helped me in clinic. They provided a foundation for the most common cases I saw.
The majority of the modules were short and to the point but had many relevant details applicable to clinic practice. It helped to understand the decision-making process.
**2. Learning and new knowledge acquisition: Did I increase my knowledge and learning as a result of doing these interactive modules?**
I felt these improved my knowledge and expanded my exposure
It could be helpful to have parts where you discuss a broad differential for various conditions (growth failure, early puberty etc.) and the types of questions you would ask to come to a diagnosis
Great to have additional a learning resource other than just reading/in-person teaching
Yes!
Yes, the concept flowed very well and made it easier to differentiate conditions
The topics were presented at a level of detail that was appropriate and an effective refresher of knowledge I gained in medical school
Yes, and I learn something every time I watch them.
Thought they were superior to textbooks
The modules hit many of the important and high-yield endocrine topics
**3. Applying the learning: Will I be able to use the information I learned from these modules when evaluating children with various conditions such as short stature**,** early or delayed puberty**,** abnormal thyroid function**,** etc?**
I felt more prepared to evaluate patients after viewing the modules.
Information in modules was high-yield and explained very well.
The modules appropriately depicted examples of patient presentations and the rationale to process them to make a diagnosis
Yes, as someone in General Pediatrics, this is very helpful
Much of applications are about repetition, and I likely won’t evaluate the aforementioned conditions particularly often. However, I feel like the modules are a great introduction to common endocrine conditions and help to reinforce knowledge.
**4. Effect on results: Do I think that the knowledge and learning will improve my effectiveness as a pediatrician?**
I feel that the modules helped prepare me for the boards and pediatric practice in general
Concepts covered in modules are great to know for any area of pediatrics
Can possibly be expanded to include more topics relevant to pediatric endocrinology
Yes, I think that these topics are important for any pediatrician who may treat patients with these conditions
Yes, especially in relation to questions about delayed growth and obesity
Yes! I will be able to explain pathology better to my patients
Helps me with screening patients in general pediatric clinic. I am more comfortable dealing with common endocrine problems, knowing the basic treatments and workups and when a referral is appropriate

## Discussion

With rising rates of diabetes, obesity, gender-affirming care, and survivorship-associated endocrinopathies among children, it is critical that the next generation of pediatricians has a strong foundation in core concepts of pediatric endocrinology, whether or not these learners go on to pursue a career in pediatric endocrinology [20, 21]. Additionally, increasing opportunities for learning in the field could contribute to a positive early experience within pediatric endocrinology for both medical students and residents, recommended by the Pediatric Endocrine Society (PES) for workforce growth and maintenance [20]. 

This study evaluated learner-reported efficacy of learner-directed CBL to augment an educational rotation through pediatric endocrinology. Implementation of these modules was associated with high learner satisfaction, perceived knowledge gains, and self-reported application to clinical practice. These findings align with prior studies supporting online CBL as an effective, engaging, and flexible educational strategy for developing clinical reasoning and knowledge retention; additionally, this work contributes to previously published studies by demonstrating CBL in a pediatric subspecialty critical for the education of a general pediatrician both for daily clinical practice and successfully passing pediatric board certification exams [7–10, 12, 22]. The flexibility and accessibility of online modules address constraints imposed by reduced protected teaching time availability of direct subspecialty clinician education, allowing residents to engage with material at their own pace and revisit challenging concepts as needed [11, 13, 14]. Importantly, this tool addresses non-diabetes topics, a needed addition given the dearth of such education in the current state of pediatric endocrinology education [21].

In 2020, the workforce taskforce of PES [20] recommended increasing positive exposures to pediatric endocrinology and diabetes during both medical school and the first year of pediatric residency to combat a growing shortage of pediatric endocrinologists amidst the backdrop of rising rates of diabetes, obesity, children seeking gender-affirming care, and endocrine consequences of other disorders (cancer and transplant-associated endocrinopathies, cystic fibrosis-related diabetes, growth failure secondary to chronic inflammatory disease, etc.). A scoping review of 45 pediatric endocrinology education publications by Alshammri et al. [21] revealed an emphasis on diabetes content and didactic strategies, with recommendations for stronger emphasis on a broader range of endocrinopathies and educational strategies, including reflection of clinical scenarios as seen in CBL.

Given that many pediatric residents may only experience pediatric endocrinology during required hospital-based core rotations (emergency medicine, inpatient wards, critical care), many online pediatric endocrinology-focused CBL programs are geared towards diabetes management. For example, Pinnaro et al. [23] developed an online CBL simulation for insulin dosing in cases of diabetic ketosis with high reported levels of resident enjoyment. DeSalvo et al. [24] created a resident curriculum with both online CBL and in-person modules to address common errors in inpatient diabetes management, yielding a significant decrease in resident errors following curriculum uptake. Gerard et al. [25] created an online CBL simulation serious game for pediatric emergency medicine students, residents, and attendings, including a case of diabetic ketoacidosis, with analysis showing content validity of the overall game. Increasing numbers of online CBL tools referencing pediatric endocrinology topics beyond diabetes have also emerged. Shenoy et al. [26] developed a CBL orientation to the outpatient pediatric endocrinology resident rotation with cases focusing on type 1 diabetes, short stature, precocious puberty, obesity, and hypothyroidism. Subsequent analyses showed significantly higher knowledge increases among those who participated in the CBL compared to those who did not, as well as statistically significant increases in confidence ratings in evaluation of type 1 diabetes, short stature, and precocious puberty [26]. Ens et al. [27] developed an online CBL tool to teach pediatric residents about precocious and delayed puberty; analyses showed improved competency and confidence in pubertal examinations among CBL participants. Davis et al. [28] developed an online CBL tool to improve the visual diagnosis in prepubertal genitourinary examinations geared towards multiple levels of medical learners (students, residents, fellows, and attendings), and their analysis showed improved performance in identification of normal variants of female anatomy. Vance et al. [29] developed online CBL modules for provision of medical care to transgender youth, adopted by students, residents and other trainees with analysis showing both knowledge gains and satisfaction. The European Society for Paediatric Endocrinology has also published an interactive online learning platform with 24 interactive CBL scenarios on a variety of endocrine disorders (including dysglycemias, dysnatremias, and disorders of growth, puberty, thyroid, adrenal, puberty, and bone) encountered in resource-limited settings [30]. Altogether, CBL programs aimed at teaching nuances of pediatric endocrinology have been found to be an effective addition to experiential learning. This study aims to add to this data by demonstrating the positive impact on and response had from such modules by trainees in common endocrinopathies. The self-directed nature of this CBL is an important aspect that may lend this strategy to more life-long learning, which is necessary for clinicians following completion of graduate medical education. This study also aims to increase this self-direction by specifically encouraging or assigning learners material to review rather than relying on the traditional module of outpatient residency rotations that assume learners are able to find material to review and do so.

Some limitations warrant consideration. The reliance on voluntary Likert responses introduces potential biases, including non-response and social desirability, the latter of which may inflate positive ratings and limit the generalizability of the educational impact [31–33]. The nature of the study relies on survey instruments, which notably in health professions education have reduced evidence of validity and reliability, especially instruments such as Likert data that can be subject to information loss and response style effects [31–33]. The questionnaire’s adaptation of the Kirkpatrick model relied on trainee predictions of their future practice with respect to levels 3 and 4 (applying learning and effect on results, respectively) as opposed to more objective assessment of trainee confidence or competency periodically following the intervention. Nonetheless, Likert data and the Kirkpatrick model remain practical tools for program evaluation in health professions education. There are also limitations regarding interpreting the actual completion of the videos, as the data is not able to differentiate whether residents completed all videos prior to completing the survey, nor when the video modules were completed in relation to time of survey completion. Finally, it is unknown why 3 eligible residents chose not to participate and whether this selection is reflective of their experience with the learning.

Additionally, the questions presented of self-perceived knowledge and impact on practice do not necessarily reflect actual behavior change or impact. Future evaluations could include the integration of objective measures of confidence and knowledge, such as pre- and post-tests or performance-based assessments (such as direct patient encounter observations) and the collection of longitudinal data to assess behavioral change, overall impact on a departmental and institutional level, and patient care outcomes. Module design could be enhanced by incorporating more interactive elements, opportunities for peer discussion, and alignment with entrustable professional activities. Learner evaluation strategies could incorporate other validated instruments and consider mixed-methods approaches to capture both quantitative and qualitative dimensions of educational impact.

## Conclusion

The aim of this study was to create online CBL educational material for resident education to bolster their experience and learning during a pediatric resident endocrinology elective. This study demonstrates that online CBL modules can enhance resident education in pediatric endocrinology by promoting active, flexible, and independent learning. Modules reinforced key competencies across patient care, medical knowledge, and practice-based learning—encouraging critical thinking and self-assessment within a virtual clinical context.

Understanding topics encountered in outpatient pediatric endocrinology is critical to general pediatric practice, as these topics reflect board-testable material, common indications for subspecialty referral, and shared understanding to ensure appropriate management of the child between the general pediatrician and their pediatric endocrinologist. By bolstering the ability of a pediatric trainee to interpret endocrinological nuances both pathological and not in such areas as thyroid function, normal variations of puberty versus abnormalities of puberty, normal variations of growth and abnormal growth, trainees can have a better understanding of what should be followed a general pediatrician and what needs referral to an endocrinologist. Given their accessibility and adaptability, similar online CBL models can be readily implemented across other pediatric and medical subspecialties to enrich learning experiences and foster deeper engagement outside of traditional clinical or didactic settings.

## Supplementary Information

Supplementary Material 1.

## Data Availability

All data generated or analyzed during this study are included in this published article and any supplementary information files.
