# Supplementary material for: Online case-based learning to enhance pediatric endocrinology resident education
Source: BMC Med Educ. 2026 Jan 8;26:206. doi: 10.1186/s12909-025-08517-5 (PMC12874685; doi:10.1186/s12909-025-08517-5)
Supplement: Supplementary file 1 — Supplementary Material 1. [file 12909_2025_8517_MOESM1_ESM.docx]

Supplementary File 1: SurveyMonkey Questionnaire. Respondents were allowed only a single Likert response of the A-D lettered options per numbered question. Free text comment space was included with each numbered question using the statement under letter E.

1. Did I enjoy the audiovisual modules?
   1. A lot
   2. Some
   3. A little
   4. None
   5. Comments (specific highlights and/or suggested improvements)
2. Did I increase my knowledge and learning as a result of doing these interactive modules?
   1. A lot
   2. Some
   3. A little
   4. None
   5. Comments (specific highlights and/or suggested improvements)
3. Will I be able to use the information I learned from these modules when evaluating children with various conditions such as short stature, early or delayed puberty, abnormal thyroid function, etc?
   1. A lot
   2. Some
   3. A little
   4. None
   5. Comments (specific highlights and/or suggested improvements)
4. Do I think that the knowledge and learning will improve my effectiveness as a pediatrician?
   1. A lot
   2. Some
   3. A little
   4. None
   5. Comments (specific highlights and/or suggested improvements)
